# Cortical excitability and the aging brain: toward a biomarker of cognitive resilience

**DOI:** 10.3389/fpsyg.2025.1542880

**Published:** 2025-02-18

**Authors:** Sara Palermo, Chiara Di Fazio, Eugenio Scaliti, Mario Stanziano, Anna Nigri, Marco Tamietto

**Affiliations:** ^1^Department of Psychology, University of Turin, Turin, Italy; ^2^Neuroradiology Unit, Diagnostic and Technology Department, Fondazione Istituto di Ricovero e Cura a Carattere Scientifico (IRCCS) Istituto Neurologico Carlo Besta, Milan, Italy; ^3^Neuroscience Institute of Turin (NIT), Turin, Italy; ^4^International School of Advanced Studies, University of Camerino, Camerino, Italy; ^5^Human Science and Technologies, University of Turin, Turin, Italy; ^6^Department of Management “Valter Cantino”, University of Turin, Turin, Italy; ^7^ALS Centre, “Rita Levi Montalcini” Department of Neuroscience, University of Turin, Turin, Italy; ^8^Department of Medical and Clinical Psychology, Tilburg University, Tilburg, Netherlands

**Keywords:** brain health, cortical excitability, cognitive resilience, aging brain, Hebbian plasticity, non-invasive brain stimulation

## Abstract

This perspective article addresses the potential use of cortical excitability (CE) as an indicator of cognitive health in aging people. Changes in CE may be considered a sign of resilience to cognitive decline in old age. The authors describe research on CE and its link to cognitive function in older adults and emphasize that it is a promising, non-invasive measure of healthy aging. They also address the current challenges in its implementation, the need for standardized measurement protocols and possible future avenues of research. If properly considered, CE could pave the way for early detection of cognitive decline and facilitate targeted interventions to promote cognitive resilience.

## 1 Introduction

In discussing the significant difference between the general life span and the more limited health span, the Brain Health Mission (Word Health Organization, 2022; Bassetti et al., 2022) emphasizes the critical need to understand the many structural and functional changes that occur as part of the complex processes of the aging brain. This endeavor highlights the importance of promoting brain health to improve not only longevity but also quality of life and recognizes that maintaining cognitive function and resilience is critical to successful aging.

Cortical excitability (CE), which refers to the tendency of the cerebral cortex to generate electrical activity in response to stimuli, holds great potential as a valuable biomarker for understanding cognitive and brain reserve (Menardi et al., 2018) (Table 1). This perspective article aims to explore the promising potential of increased CE levels compared to baseline measures or normative aging profiles, as a reliable clinical indicator of resilience in the elderly. Ultimately, this article aims to present compelling evidence for the link between the brain’s remarkable adaptive capacity in late life and age-related cognitive abilities.

**Table 1:** Glossary of terms.

Term	Definition	Focus
Brain reserve	The brain’s structural capacity to tolerate physical damage without showing clinical symptoms. Based on factors like brain volume and neuronal density	Physical structure and structural integrity
Brain resilience	The brain’s capacity to actively adapt and respond to stress, injury, or disease, maintaining brain function	Dynamic adaptation; cellular and molecular repair processes
Cortical excitability	The responsiveness of cortical neurons to stimuli, reflecting the brain ability to adapt neural activity in response to changing conditions	Measurement of neural responsiveness, available as a biomarker for neural function and health
Hebbian plasticity	A type of synaptic plasticity where simultaneous activation of neurons strengthens the synapse, encapsulated by the phrase “cells the fire together, wire together”	Mechanism for learning and memory, critical in adaptive neural and cognitive responses
Cognitive reserve	The brain’s ability to use alternative cognitive strategies based on know what-know-how to maintain performance despite damage or aging	Compensatory strategies and cognitive resources developed over time (e.g. experiences, education)
Cognitive resilience	The capacity of cognition to adapt and maintain functionality in face of stressors and changes, through mechanisms like synaptic plasticity and cognitive flexibility	Real-time cognitive adaptation to stress, preserving cognitive performance under challenge

Building on this theoretical framework, the researchers envision a future in which a deeper understanding of CE and its potential as a biomarker can drive the development of targeted preventive interventions. By harnessing this knowledge, clinicians and researchers can empower older adults to age healthily and maintain their overall well-being and quality of life.

### 1.1 Understanding cognitive resilience in old age

As people are living longer, maintaining cognitive function in old age is becoming increasingly important. Cognitive resilience—the ability of the mind to adapt and compensate for challenges—has been shown to be a key factor in protecting against age-related cognitive decline and neuropathologies. Cognitive resilience is distinct from brain resilience, which is about the brain’s structural ability to withstand or recover from injury and disease (Joshi and Galvin, 2022; de Vries et al., 2024; Stern et al., 2023). Cognitive resilience instead emphasizes mental flexibility and adaptive strategies that help maintain cognitive performance over time (Table 1). Given the natural decline in neural systems and overall health as we age, research on cognitive resilience provides valuable insights into how older adults can maintain their autonomy and improve their quality of life, which could reduce the healthcare costs associated with cognitive impairment (Prince et al., 2024).

Research has identified several factors that contribute to cognitive resilience, such as education, social engagement and lifestyle that include physical activity and mental stimulation (Valenzuela and Sachdev, 2006; Díaz-Venegas et al., 2019). However, much remains to be understood about how these factors interact to promote cognitive health. Emerging areas of interest include the role of cortical arousal patterns, which may offer new insights into strengthening cognitive resilience and provide opportunities for targeted interventions to promote cognitive health throughout the aging process.

### 1.2 Rationale for examining cortical excitability as a biomarker

The development of reliable biomarkers to assess cognitive resilience and predict cognitive decline has become an urgent priority in aging research (Stern et al., 2023; Stern et al., 2019). Current genetic and imaging markers show strong associations with age-related cognitive decline, but their high cost and complexity make them impractical for comprehensive screening in the general population (Shen et al., 2014; Dartora et al., 2021; Lancione et al., 2022). In contrast, CE—the tendency of the cerebral cortex to generate electrical responses to stimuli (Valenzuela and Sachdev, 2006)—is proving to be a cheaper, more accessible and plausible biomarker for assessing cognitive health for primary preventive initiatives (Menardi et al., 2018; Costanzo et al., 2024; Pellegrino et al., 2024).

As we age, neurophysiological modification, such as changes in neurotransmitter concentration, synaptic strength and ion channel function, can affect CE (Menardi et al., 2018). Elevated CE levels have been consistently associated with poorer cognitive function and may indicate an increased risk of age-related pathophysiological changes, including neuronal degeneration (Menardi et al., 2018; Buss et al., 2023; Chou et al., 2022). This association highlights CE as a sensitive indicator of brain health that has the potential to detect subtle changes in neuronal function before overt cognitive decline occurs.

CE is closely related to Hebbian plasticity—the brain’s adaptive ability to reorganize and strengthen synaptic connections in response to experience (von Bernhardi et al., 2017; Magee and Grienberger, 2020). Since Hebbian plasticity is essential for learning and memory, CE can serve as an indicator of the brain’s adaptive potential, which is crucial for cognitive resilience (Menardi et al., 2018). By studying how CE changes with age, researchers can gain insight into the brain’s ability to maintain cognitive performance and adapt to age-related stressors (Menardi et al., 2022).

In addition, CE provides a unique insight into the balance of excitatory and inhibitory processes in the brain. Disruptions to this balance are often observed in age-related cognitive decline, which is characterized by increased neuronal noise and decreased signal-to-noise ratio. By assessing CE, it becomes possible to assess how excitability and inhibition dynamics evolve with aging and how they affect cognitive resilience (Cespón et al., 2022). This could support the development of targeted interventions to restore neuronal balance and improve cognitive health (Menardi et al., 2022; Cordeiro et al., 2024).

Another compelling reason for investigating CE as a biomarker lies in its non-invasive measurement using established techniques such as non-invasive brain stimulation (NIBS) in combination with electroencephalography (EEG) (Menardi et al., 2018; Pellegrino et al., 2024). These methods enable precise real-time assessment of cortical responses and are therefore suitable for both longitudinal studies and clinical application. Unlike complex imaging techniques, they provide a practical means of monitoring and promoting cognitive health in large populations, which fits well with public health goals focusing on early detection and preventive care.

Finally, the integration of CE as a biomarker is in line with initiatives such as the Brain Health Mission (Word Health Organization, 2022; Bassetti et al., 2022), which emphasize extending life in good health rather than just extending lifespan. The identification of practical and reliable biomarkers such as CE supports proactive measures to promote cognitive health, reduce age-related neuropathologies and ultimately improve the quality of life of older people.

## 2 Cortical excitability and age-related changes

CE is also emerging as a promising biomarker for assessing the health of the sensorimotor system, which may provide insight into cognitive resilience in old age. For example, a reduction in CE may improve the brain’s signal-to-noise ratio, aid cognitive processing and potentially mitigate cognitive decline (Al et al., 2023). As a measure of the ability of the neuronal membrane to generate action potentials, CE can be measured using various techniques that provide insight into how age affects communication between different neuronal networks (Li et al., 2015; Badawy et al., 2012; Ortega-Robles et al., 2023). Each network—e.g., sensory-motor, visual, auditory or the default mode network—contributes to cognitive function in a unique way, and aging affects these networks differently in each person (Dubbioso et al., 2019; Hui et al., 2023; Peng et al., 2024; Dinse et al., 2023). For example, the sensorimotor network may suffer greater age-related effects in some people (Li et al., 2015; Cassady et al., 2019), affecting motor perception and speech modulation, while others may experience pronounced changes in the visual or auditory network, affecting sensory processing (Yoshimura et al., 2020; Zhang et al., 2023). The default mode network, which is responsible for brain activity at rest, also shows different age-related susceptibility, possibly affecting memory and attention. The interaction of CE with these networks is thus crucial to determining its role in cognitive aging (Gonzalez-Escamilla et al., 2018). Studies that map CE in different networks can help clarify the link between CE and specific cognitive functions and how these are preserved or decline with age. The aging brain is characterized by several cellular and structural changes, collectively referred to as neurodegeneration, which include shifts in gray and white matter as well as changes in CE (Chou et al., 2022; Lu et al., 2024). Research shows that CE generally declines with age, although this decline can vary greatly depending on brain reserve, brain physiology and cognitive reserve (Pettigrew and Soldan, 2019; Savarimuthu and Ponniah, 2024). In the pathological cognitive aging, reduced CE is observed, suggesting that this may be a compensatory mechanism for the progressive loss of cortical neurons. This decline continues until the advanced stages of the disease, when a critical point of cortical atrophy is reached. It is hypothesized that the gradual change in the relationship between reduced CE and cognitive performance reflects the point at which hyperexcitability ceases to be compensatory and becomes detrimental to individuals experiencing cognitive impairments, due to an increasing impediment in the allocation of cognitive resources (Peng et al., 2024; Xie et al., 2023). The cognitive reserve serves as a means to prolong functioning and delay reaching this critical point (Menardi et al., 2018; Tagliabue and Mazza, 2021).

In this context, Non-invasive brain stimulation techniques, such as transcranial magnetic stimulation (TMS) have emerged as a potential tool to assess CE as a “malleable” biomarker that is sensitive to age and possibly cognitive health (Tremblay et al., 2019; Kallioniemi and Daskalakis, 2022; Williams et al., 2021). Both over-excitation and under-excitation of cortical populations have been associated with specific neuropathologies, emphasizing the importance of neuronal health in interpreting measures of excitability in relation to cognitive aging (Pagali et al., 2024). TMS offers a promising approach to assess CE across the lifespan, but its application requires an evolving understanding of excitability as a dynamic construct that changes with age (Motta et al., 2018; Ferreri et al., 2021). Integrating findings from TMS and other modalities could ultimately improve our understanding of CE as a valuable biomarker of cognitive aging and promote a comprehensive approach to promoting cognitive resilience in the aging brain (Menardi et al., 2018; Motta et al., 2018; Ferreri et al., 2021).

## 3 Cortical excitability and cognitive performance in older adults

**Figure 1:**
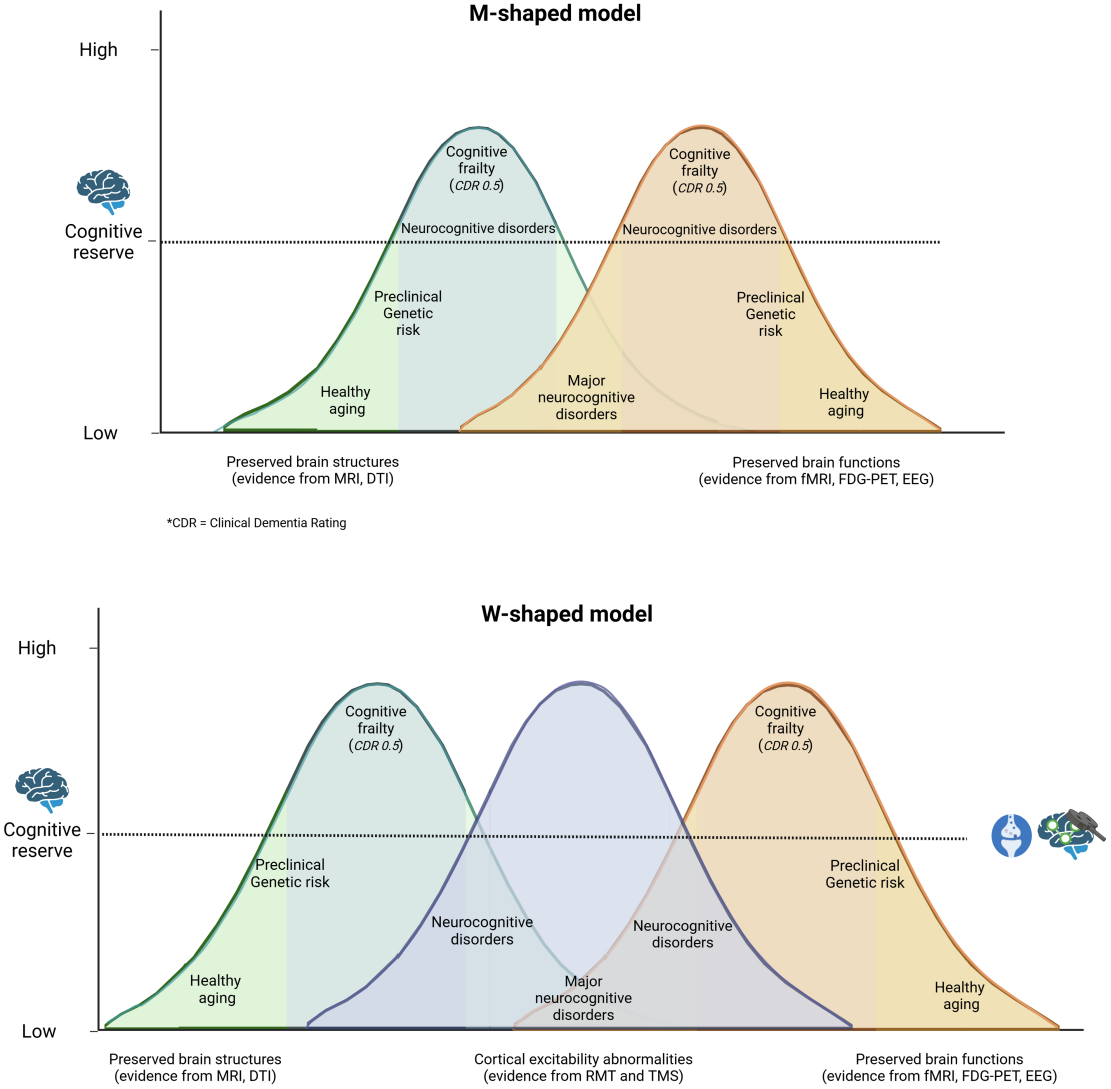
Traditionally, it has been hypothesized that cognitive reserve protects the individual by delaying the onset of symptoms and the time of clinical diagnosis. A M-shaped model reflects a more complex reality in which both individuals who age successfully and those who are on the pathway from normal cognitive aging to mild/major neurocognitive disorder exhibit cognitive impairment and gradual but opposing structural and functional changes in the central nervous system, depending on cognitive reserve. A more plausible model might be W-shaped, where altered cortical excitability is seen as a measure that encompasses structural and functional changes in the brain. This illustrates the potential implementation of non-invasive brain stimulation (NIBS) in clinical practice to assess an individual’s placement on the health-pathology continuum (Menardi et al., 2018).

A growing body of evidence suggests that CE plays a crucial role in cognitive performance in older people (Cespón et al., 2022; Tang et al., 2019). Memory, attention, working memory and executive functions have been linked to CE (Xu et al., 2024; Mansouri et al., 2015). These correlations reflect the presence of an optimal level of arousal, below and above which cognitive functions are impaired. This correspondence between cognitive functions and the level of CE can be understood in the context of the inverted-U hypothesis of excitability in memory and learning tasks (Menardi et al., 2018; Zadey et al., 2021; Baldi and Bucherelli, 2005), as shown in Figure 1. Two important lines of research linking CE to cognition are the investigation of whether excitability covaries with cognitive performance in different individuals and the investigation of whether the level of excitability can predict longitudinal changes in cognitive performance during aging (Dinse et al., 2023; Zadey et al., 2021). The relationship between CE and cognitive performance makes it a prime candidate for a potential biomarker of cognitive performance in general and cognitive reserve in particular (Cespón et al., 2022; Lu et al., 2024; Berns et al., 2020).

In several studies of older adults without cognitive impairments, a large variation in cognitive function between individuals has been observed, which is associated with a large variation in the level of CE (Buss et al., 2023; Zadey et al., 2021; Van Egroo et al., 2019). Older adults with preserved robust cognitive performance show lower CE compared to less cognitively robust older adults, suggesting that their lower excitability is associated with maximal cognitive function (Cespón et al., 2022). To date, no study has been able to prospectively link changes in CE to individual changes in cognitive function in healthy older people across the lifespan. Risk factors that increase the occurrence of cognitive impairment, such as amyloid deposition or entropy load, are expected to increase the level of excitability, which according to the inverted-U hypothesis will lead to a decline in cognitive function (Targa Dias Anastacio et al., 2022). This provides an ideal opportunity to develop individualized predictive markers. To summarize, there is growing evidence that CE can provide insight into which individuals are at risk of cognitive impairment and could be used as an early detection tool to enable early intervention before cognitive impairment occurs in older adults (Cantone et al., 2023; Sharbafshaaer et al., 2023). Preliminary evidence suggests that CE is reduced in the commonly observed cases of cognitive resilience in old age (Buss et al., 2023). CE could therefore be a marker for this type of diversity in resilience, i.e., for defensive and proactive strategies to prevent pathological consequences of cognitive aging (Gonzalez-Escamilla et al., 2018).

## 4 The use of cortical excitability as a biomarker and potential intervention target: promise and challenges

Preventive strategies targeting cognitive aging are particularly promising as they are low-risk, non-invasive and cost-effective (Livingston et al., 2020). By characterizing individual CE profiles, researchers can better understand how these markers predict, track and potentially even reverse accelerated cognitive decline (Stern, 2006; Stern, 2009). This finding supports their role as valuable public health strategies for maintaining cognitive health and quality of life in older adults. In particular, decreasing excitability has been proposed as a neural mechanism underlying accelerated cognitive decline and neuropathological changes (World Health Organization, 2017). This emphasizes the importance of identifying individuals with atypical changes in CE, cognition or neuronal volume for targeted interventions.

This relationship aligns with the inverted-U hypothesis illustrated in Figure 1, which emphasizes that both excessive and diminished cortical excitability (CE) can result in maladaptive cognitive outcomes. Elevated CE levels, for instance, could reflect compensatory mechanisms in healthy aging or dysregulated excitatory-inhibitory dynamics in pathological aging. This suggests that interventions should be customized based on individual excitability patterns to achieve the best possible results.

Expanding further, direct methods such as brain stimulation and exercise offer promising avenues for enhancing CE. Neuromodulation therapies, including TMS, provide non-invasive and highly targeted means of altering neural network activity (Borgomaneri et al., 2022; Borgomaneri et al., 2021; Borgomaneri et al., 2020). Research shows that single-pulse TMS can improve task performance, speed and memory in older people, with these benefits correlating with baseline excitability (Ortega-Robles et al., 2023; Thut et al., 2003; Miniussi and Thut, 2010). Higher CE induced by TMS has been associated with faster processing speed and improved executive function, although the effects are often task-dependent and vary with cognitive load and task type (Menardi et al., 2018; Buss et al., 2023; Menardi et al., 2022).

Increasing CE has implications for cognitive resilience. Higher CE levels could support cognitive reserve by improving the brain’s flexibility in managing task-relevant resources, which could attenuate age-related cognitive differences (Stern et al., 2019; Stern, 2009; Stern, 2012). In this sense, individuals with higher CE might have a greater “cognitive resource capacity” that allows them to adapt more effectively to cognitive demands (Menardi et al., 2018; Menardi et al., 2022). However, measuring neurobiological variability using CE parameters in older adults remains challenging due to differences in methods, measurement reliability and participant characteristics (Mattay et al., 2008; Kramer and Colcombe, 2018; Kivipelto et al., 2018). Advances in test–retest reliability now support more accurate assessments, allowing researchers to track individual differences in neural physiology with greater precision (Valenzuela and Sachdev, 2006; Zadey et al., 2021; Kivipelto et al., 2018).

The potential of CE as a biomarker of cognitive reserve emphasizes the need for an interdisciplinary approach that combines cognitive neuroscience, gerontology and neurophysiology. Aligning conceptual frameworks and research methodologies between the different fields will be critical to advancing this burgeoning field and enabling more effective integration of cognitive reserve theory into brain health initiatives (Valenzuela and Sachdev, 2006; Livingston et al., 2020; Mattay et al., 2008; Raz et al., 1998). As this field matures, it presents an exciting opportunity to deepen our understanding of aging, promote cognitive resilience, and develop new prevention strategies for cognitive health.

## 5 Discussion

The authors suggest that corticomotor excitability may serve as a dynamic biomarker of cognitive resilience, a concept that is more and more investigated in studies of healthy aging and for which there is considerable theoretical overlap in brain and cognitive aging research (Menardi et al., 2018; Buss et al., 2023).

CE is particularly promising because it is easily accessible, reflects the health of neural circuits, can be measured non-invasively, and is related to neuronal plasticity and the balance between excitation and inhibition (Buss et al., 2023; Cespón et al., 2022; Dinse et al., 2023; Meder et al., 2021). As research progresses, CE could play an important role in the early detection of cognitive impairment and lead to timely interventions that promote cognitive resilience and support healthy aging.

The role of cognitive resilience in maintaining cognitive health during aging emphasizes the importance of further research into these mechanisms (Joshi and Galvin, 2022; Stern et al., 2023). The variability of changes in CE across individuals highlights the importance of not only observing baseline data, but also tracking how it relates to longitudinal cognitive or sensorimotor changes (de Vries et al., 2024; Stern et al., 2019; Cordeiro et al., 2024; Gonzalez-Escamilla et al., 2018). Moreover, the question of how the modulation of CE influences cognitive resilience should be investigated in more detail. The knowledge gained from this research could enable the development of new strategies to optimize cognitive function, enhance well-being and improve quality of life in old age.

Future approaches could combine cognitive training with non-invasive brain stimulation techniques to increase CE. Refining models of cortical excitability, such as the proposed ‘W’ framework, could help identify the balance between compensatory and maladaptive states, guiding more targeted interventions. The use of individual profiles of neuronal plasticity could further personalize these interventions and maximize their effectiveness. Advances in neuroscience methods that allow real-time monitoring of excitability changes could enable a more comprehensive approach to cognitive health, integrating CE with cognitive reserve metrics to better predict and mitigate age-related cognitive decline while differentiating between under- and over-excitability in aging individuals.

Personalized interventions are particularly promising due to their excellent safety profile, so these new methods and neurobiological findings represent a potential breakthrough for public health and scientific progress. The improved ability to create individual profiles of the aging brain could pave the way for a new, personalized paradigm of cognitive resilience interventions and ultimately benefit the cognitive health of the population.

## Data Availability

The original contributions presented in the study are included in the article/supplementary material, further inquiries can be directed to the corresponding author.

## References

[ref1] Al E. Stephani T. Engelhardt M. Haegens S. Villringer A. Nikulin V. V. (2023). Cardiac activity impacts cortical motor excitability. PLoS Biol. 21:e3002393. doi: 10.1371/journal.pbio.3002393, PMID: 38015826 PMC10684011

[ref2] Badawy R. A. Loetscher T. Macdonell R. A. L. Brodtmann A. (2012). Cortical excitability and neurology: insights into the pathophysiology. Funct. Neurol. 27, 131–145, PMID: 23402674 PMC3812767

[ref3] Baldi E. Bucherelli C. (2005). The inverted “U-shaped” dose-effect relationships in learning and memory: modulation of arousal and consolidation. Nonlinearity Biol. Toxicol. Med. 3, 9–21. doi: 10.2201/nonlin.003.01.002, PMID: 19330154 PMC2657842

[ref4] Bassetti C. L. A. Endres M. Sander A. Crean M. Subramaniam S. Carvalho V.. (2022). The European academy of neurology brain health strategy: one brain, one life, one approach. Eur. J. Neurol. 29, 2559–2566. doi: 10.1111/ene.15391, PMID: 35538709

[ref5] Berns C. Brüchle W. Scho S. Schneefeld J. Schneider U. Rosenkranz K. (2020). Intensity dependent effect of cognitive training on motor cortical plasticity and cognitive performance in humans. Exp. Brain Res. 238, 2805–2818. doi: 10.1007/s00221-020-05933-5, PMID: 33025030 PMC7644474

[ref6] Borgomaneri S. Battaglia S. Avenanti A. di Pellegrino G. (2021). Don’t hurt me no more: state-dependent transcranial magnetic stimulation for the treatment of specific phobia. J. Affect. Disord. 286, 78–79. doi: 10.1016/j.jad.2021.02.076, PMID: 33714173

[ref7] Borgomaneri S. Battaglia S. Garofalo S. Tortora F. Avenanti A. di Pellegrino G. (2020). State-dependent TMS over prefrontal cortex disrupts fear-memory reconsolidation and prevents the return of fear. Curr. Biol. 30, 3672–3679.e4. doi: 10.1016/j.cub.2020.06.091, PMID: 32735813

[ref8] Borgomaneri S. Zanon M. Di Luzio P. Romei V. Tamietto M. Avenanti A. (2022). Driving associative plasticity in temporo-occipital back-projections improves visual recognition of emotional expressions. Nat. Commun. 14:5720. doi: 10.1038/s41467-023-41058-3 PMC10517146 37737239

[ref9] Buss S. S. Fried P. J. Macone J. Zeng V. Zingg E. Santarnecchi E.. (2023). Greater cognitive reserve is related to lower cortical excitability in healthy cognitive aging, but not in early clinical Alzheimer’s disease. Front. Hum. Neurosci. 17:1193407. doi: 10.3389/fnhum.2023.1193407, PMID: 37576473 PMC10413110

[ref10] Cantone M. Fisicaro F. Ferri R. Bella R. Pennisi G. Lanza G.. (2023). Sex differences in mild vascular cognitive impairment: a multimodal transcranial magnetic stimulation study. PLoS One 18:e0282751. doi: 10.1371/journal.pone.0282751, PMID: 36867595 PMC9983846

[ref11] Cassady K. Gagnon H. Lalwani P. Simmonite M. Foerster B. Park D.. (2019). Sensorimotor network segregation declines with age and is linked to GABA and to sensorimotor performance. NeuroImage 186, 234–244. doi: 10.1016/j.neuroimage.2018.11.008, PMID: 30414983 PMC6338503

[ref12] Cespón J. Pellicciari M. C. Casula E. P. Miniussi C. (2022). Age-related changes in cortical excitability linked to decreased attentional and inhibitory control. Neuroscience 495, 1–14. doi: 10.1016/j.neuroscience.2022.05.021 35605905

[ref13] Chou Y. H. Sundman M. Ton That V. Greena J. Trapani C. (2022). Cortical excitability and plasticity in Alzheimer’s disease and mild cognitive impairment: A systematic review and meta-analysis of transcranial magnetic stimulation studies. Ageing Res. Rev. 79:101660. doi: 10.1016/j.arr.2022.101660 35680080 PMC9707650

[ref14] Cordeiro A. Gomes C. Bicker J. Fortuna A. (2024). Aging and cognitive resilience: Molecular mechanisms as new potential therapeutic targets. Drug Discov. Today 29:104093. doi: 10.1016/j.drudis.2024.104093 38992420

[ref15] Costanzo M. Cutrona C. Leodori G. Malimpensa L. D’antonio F. Conte A.. (2024). Exploring easily accessible neurophysiological biomarkers for predicting Alzheimer’s disease progression: a systematic review. Alzheimers Res. Ther. 16:244. doi: 10.1186/s13195-024-01607-4, PMID: 39497149 PMC11533378

[ref16] Dartora C. M. Borelli W. V. Koole M. Marques da Silva A. M. (2021). Cognitive decline assessment: A review from medical imaging perspective. Front. Aging Neurosci. 13:704661. doi: 10.3389/fnagi.2021.704661 34489675 PMC8416532

[ref17] de Vries L. E. Huitinga I. Kessels H. W. Swaab D. F. Verhaagen J. (2024). The concept of resilience to Alzheimer’s disease: current definitions and cellular and molecular mechanisms. Mol. Neurodegener. 19:33. doi: 10.1186/s13024-024-00719-7, PMID: 38589893 PMC11003087

[ref18] Díaz-Venegas C. Samper-Ternent R. Michaels-Obregón A. Wong R. (2019). The effect of educational attainment on cognition of older adults: results from the Mexican health and aging study 2001 and 2012. Aging Ment. Health 23, 1586–1594. doi: 10.1080/13607863.2018.1501663, PMID: 30449138 PMC6525654

[ref19] Dinse H. R. Höffken O. Tegenthoff M. (2023). Cortical excitability in human somatosensory and visual cortex: Implications for plasticity and learning – A minireview. Front. Hum. Neurosci. 17:1235487. doi: 10.3389/fnhum.2023.1235487 37662638 PMC10469727

[ref20] Dubbioso R. Manganelli F. Siebner H. R. Di Lazzaro V. (2019). Fast intracortical sensory-motor integration: a window into the pathophysiology of parkinson’s disease. Front. Hum. Neurosci. 13:13. doi: 10.3389/fnhum.2019.00111, PMID: 31024277 PMC6463734

[ref21] Ferreri F. Guerra A. Vollero L. Ponzo D. Määtta S. Könönen M.. (2021). TMS-EEG biomarkers of amnestic mild cognitive impairment due to Alzheimer’s disease: a proof-of-concept six years prospective study. Front. Aging Neurosci. 13:13. doi: 10.3389/fnagi.2021.737281, PMID: 34880743 PMC8645846

[ref22] Gonzalez-Escamilla G. Muthuraman M. Chirumamilla V. C. Vogt J. Groppa S. (2018). Brain networks reorganization during maturation and healthy aging-emphases for resilience. Front. Psychiatry 9:601. doi: 10.3389/fpsyt.2018.00601 30519196 PMC6258799

[ref23] Hui C. L. M. Wong S. M. Y. Yu T. Y. T. Lau T. T. Y. Choi O. Tsang S.. (2023). Visual-stress-related cortical excitability as a prospective marker for symptoms of depression and anxiety in young people. Eur. Arch. Psychiatry Clin. Neurosci. 273, 1051–1060. doi: 10.1007/s00406-022-01469-7, PMID: 35972556

[ref24] Joshi M. S. Galvin J. E. (2022). Cognitive resilience in brain health and dementia research. J. Alzheimer’s Dis. 90, 461–473. doi: 10.3233/JAD-220755 36093713 PMC10515194

[ref25] Kallioniemi E. Daskalakis Z. J. (2022). Identifying novel biomarkers with TMS-EEG – methodological possibilities and challenges. J. Neurosci. Methods 377:109631. doi: 10.1016/j.jneumeth.2022.109631, PMID: 35623474

[ref26] Kivipelto M. Mangialasche F. Ngandu T. (2018). Lifestyle interventions to prevent cognitive impairment, dementia and Alzheimer disease. Nat. Rev. Neurol. 14, 653–666. doi: 10.1038/s41582-018-0070-3 30291317

[ref27] Kramer A. F. Colcombe S. (2018). Fitness effects on the cognitive function of older adults: a Meta-analytic study—revisited. Perspect. Psychol. Sci. 13, 213–217. doi: 10.1177/1745691617707316 29592650

[ref28] Lancione M. Bosco P. Costagli M. Nigri A. Aquino D. Carne I.. (2022). Multi-Centre and multi-vendor reproducibility of a standardized protocol for quantitative susceptibility mapping of the human brain at 3T. Phys. Med. 103, 37–45. doi: 10.1016/j.ejmp.2022.09.012, PMID: 36219961

[ref29] Li H. J. Hou X. H. Liu H. H. Yue C. L. Lu G. M. Zuo X. N. (2015). Putting age-related task activation into large-scale brain networks: a meta-analysis of 114 fMRI studies on healthy aging. Neurosci. Biobehav. Rev. 57, 156–174. doi: 10.1016/j.neubiorev.2015.08.013, PMID: 26318367

[ref30] Livingston G. Huntley J. Sommerlad A. Ames D. Ballard C. Banerjee S.. (2020). Dementia prevention, intervention, and care: 2020 report of the lancet commission. Lancet 396, 413–446. doi: 10.1016/S0140-6736(20)30367-6 32738937 PMC7392084

[ref31] Lu Q. Huang S. Zhang T. Song J. Dong M. Qian Y.. (2024). Age-related differences in long-term potentiation-like plasticity and short latency afferent inhibition and their association with cognitive function. Gen Psychiatr. 37:e101181. doi: 10.1136/gpsych-2023-101181, PMID: 38390239 PMC10882289

[ref32] Magee J. C. Grienberger C. (2020). Synaptic plasticity forms and functions. Annu. Rev. Neurosci. 43, 95–117. doi: 10.1146/annurev-neuro-090919-32075520

[ref33] Mansouri F. A. Rosa M. G. P. Atapour N. (2015). Working memory in the service of executive control functions. Front. Syst. Neurosci. 9:166. doi: 10.3389/fnsys.2015.00166 26696841 PMC4677100

[ref34] Mattay V. S. Goldberg T. E. Sambataro F. Weinberger D. R. (2008). Neurobiology of cognitive aging: insights from imaging genetics. Biol. Psychol. 79, 9–22. doi: 10.1016/j.biopsycho.2008.03.015, PMID: 18511173 PMC3127547

[ref35] Meder A. Liepelt-Scarfone I. Sulzer P. Berg D. Laske C. Preische O.. (2021). Motor cortical excitability and paired-associative stimulation-induced plasticity in amnestic mild cognitive impairment and Alzheimer’s disease. Clin. Neurophysiol. 132, 2264–2273. doi: 10.1016/j.clinph.2021.01.011, PMID: 33612394

[ref36] Menardi A. Pascual-Leone A. Fried P. J. Santarnecchi E. (2018). The role of cognitive Reserve in Alzheimer’s disease and aging: a multi-modal imaging review. J. Alzheimer’s Dis. 66, 1341–1362. doi: 10.3233/JAD-180549, PMID: 30507572 PMC8972845

[ref37] Menardi A. Rossi S. Koch G. Hampel H. Vergallo A. Nitsche M. A.. (2022). Toward noninvasive brain stimulation 2.0 in Alzheimer’s disease. Ageing Res. Rev. 75:101555. doi: 10.1016/j.arr.2021.101555, PMID: 34973457 PMC8858588

[ref38] Miniussi C. Thut G. (2010). Combining TMS and EEG offers new prospects in cognitive neuroscience. Brain Topogr. 22, 249–256. doi: 10.1007/s10548-009-0083-8, PMID: 19241152

[ref39] Motta C. Di Lorenzo F. Ponzo V. Pellicciari M. C. Bonnì S. Picazio S.. (2018). Transcranial magnetic stimulation predicts cognitive decline in patients with Alzheimer’s disease. J. Neurol. Neurosurg. Psychiatry 89, 1237–1242. doi: 10.1136/jnnp-2017-317879, PMID: 30464028

[ref40] Ortega-Robles E. Cantillo-Negrete J. Carino-Escobar R. I. Arias-Carrión O. (2023). Methodological approach for assessing motor cortical excitability changes with single-pulse transcranial magnetic stimulation. MethodsX 11:102451. doi: 10.1016/j.mex.2023.102451, PMID: 38023316 PMC10630640

[ref41] Pagali S. R. Kumar R. LeMahieu A. M. Basso M. R. Boeve B. F. Croarkin P. E.. (2024). Efficacy and safety of transcranial magnetic stimulation on cognition in mild cognitive impairment, Alzheimer’s disease, Alzheimer’s disease-related dementias, and other cognitive disorders: A systematic review and meta-analysis. Int. Psychogeriatr. 36, 880–928. doi: 10.1017/S1041610224000085, PMID: 38329083 PMC11306417

[ref42] Pellegrino G. Schuler A. L. Cai Z. Marinazzo D. Tecchio F. Ricci L.. (2024). Assessing cortical excitability with electroencephalography: a pilot study with EEG-iTBS. Brain Stimul. 17, 176–183. doi: 10.1016/j.brs.2024.01.004, PMID: 38286400

[ref43] Peng J. Zikereya T. Shao Z. Shi K. (2024). The neuromechanical of Beta-band corticomuscular coupling within the human motor system. Front. Neurosci. 18:1441002. doi: 10.3389/fnins.2024.1441002 39211436 PMC11358111

[ref44] Pettigrew C. Soldan A. (2019). Defining cognitive reserve and implications for cognitive aging. Curr. Neurol. Neurosci. Rep. 19:1. doi: 10.1007/s11910-019-0917-z 30627880 PMC7812665

[ref45] Prince J. B. Davis H. L. Tan J. Muller-Townsend K. Markovic S. Lewis D. M. G.. (2024). Cognitive and neuroscientific perspectives of healthy ageing. Neurosci. Biobehav. Rev. 161:105649. doi: 10.1016/j.neubiorev.2024.105649 38579902

[ref46] Raz N. Gunning-Dixon F. M. Head D. Dupuis J. H. Acker J. D. (1998). Neuroanatomical correlates of cognitive aging: evidence from structural magnetic resonance imaging. Neuropsychology 12, 95–114. doi: 10.1037/0894-4105.12.1.95 9460738

[ref47] Savarimuthu A. Ponniah R. J. (2024). Cognition and cognitive reserve. Integr. Psychol. Behav. Sci. 58, 483–501. doi: 10.1007/s12124-024-09821-3, PMID: 38279076

[ref48] Sharbafshaaer M. Gigi I. Lavorgna L. Esposito S. Bonavita S. Tedeschi G.. (2023). Repetitive transcranial magnetic stimulation (rTMS) in mild cognitive impairment: Effects on cognitive functions—A systematic review. J. Clin. Med. 12:6190. doi: 10.3390/jcm12196190 37834834 PMC10573645

[ref49] Shen L. Thompson P. M. Potkin S. G. Bertram L. Farrer L. A. Foroud T. M.. (2014). Genetic analysis of quantitative phenotypes in AD and MCI: imaging, cognition and biomarkers. Brain Imaging Behav. 8, 183–207. doi: 10.1007/s11682-013-9262-z, PMID: 24092460 PMC3976843

[ref50] Stern Y. (2006). Cognitive reserve and Alzheimer disease. Alzheimer Dis. Assoc. Disord. 20, 112–117. doi: 10.1097/01.wad.0000213815.20177.19 16772747

[ref51] Stern Y. (2009). Cognitive reserve. Neuropsychologia 47, 2015–2028. doi: 10.1016/j.neuropsychologia.2009.03.004 19467352 PMC2739591

[ref52] Stern Y. (2012). Cognitive reserve in ageing and Alzheimer’s disease. Lancet Neurol. 11, 1006–1012. doi: 10.1016/S1474-4422(12)70191-6, PMID: 23079557 PMC3507991

[ref53] Stern Y. Albert M. Barnes C. A. Cabeza R. Pascual-Leone A. Rapp P. R. (2023). A framework for concepts of reserve and resilience in aging. Neurobiol. Aging 124, 100–103. doi: 10.1016/j.neurobiolaging.2022.10.015 36653245 PMC10424718

[ref54] Stern Y. Barnes C. A. Grady C. Jones R. N. Raz N. (2019). Brain reserve, cognitive reserve, compensation, and maintenance: operationalization, validity, and mechanisms of cognitive resilience. Neurobiol. Aging 83, 124–129. doi: 10.1016/j.neurobiolaging.2019.03.022, PMID: 31732015 PMC6859943

[ref55] Tagliabue C. F. Mazza V. (2021). What can neural activity tell us about cognitive resources in aging? Front. Psychol. 12:753423. doi: 10.3389/fpsyg.2021.753423 34733219 PMC8558238

[ref56] Tang X. Huang P. Li Y. Lan J. Yang Z. Xu M.. (2019). Age-related changes in the plasticity of neural networks assessed by transcranial magnetic stimulation with electromyography: A systematic review and Meta-analysis. Front. Cell. Neurosci. 13:469. doi: 10.3389/fncel.2019.00469 31708744 PMC6822534

[ref57] Targa Dias Anastacio H. Matosin N. Ooi L. (2022). Neuronal hyperexcitability in Alzheimer’s disease: what are the drivers behind this aberrant phenotype? Transl. Psychiatry 12:257. doi: 10.1038/s41398-022-02024-7 35732622 PMC9217953

[ref58] Thut G. Northoff G. Ives J. R. Kamitani Y. Pfennig A. Kampmann F.. (2003). Effects of single-pulse transcranial magnetic stimulation (TMS) on functional brain activity: a combined event-related TMS and evoked potential study. Clin. Neurophysiol. 114, 2071–2080. doi: 10.1016/S1388-2457(03)00205-0, PMID: 14580605

[ref59] Tremblay S. Rogasch N. C. Premoli I. Blumberger D. M. Casarotto S. Chen R.. (2019). Clinical utility and prospective of TMS–EEG. Clin. Neurophysiol. 130, 802–844. doi: 10.1016/j.clinph.2019.01.001 30772238

[ref60] Valenzuela M. J. Sachdev P. (2006). Brain reserve and cognitive decline: A non-parametric systematic review. Psychol. Med. 36, 1065–1073. doi: 10.1017/S0033291706007744 16650343

[ref61] Van Egroo M. Narbutas J. Chylinski D. Villar González P. Ghaemmaghami P. Muto V.. (2019). Preserved wake-dependent cortical excitability dynamics predict cognitive fitness beyond age-related brain alterations. Commun. Biol. 2:449. doi: 10.1038/s42003-019-0693-y, PMID: 31815203 PMC6890637

[ref62] von Bernhardi R. von Bernhardi L. E. Eugenín J. (2017). What is neural plasticity? Adv. Exp. Med. Biol. 1015, 1–15. doi: 10.1007/978-3-319-62817-2_1, PMID: 29080018

[ref63] Williams L. M. Coman J. T. Stetz P. C. Walker N. C. Kozel F. A. George M. S.. (2021). Identifying response and predictive biomarkers for transcranial magnetic stimulation outcomes: protocol and rationale for a mechanistic study of functional neuroimaging and behavioral biomarkers in veterans with Pharmacoresistant depression. BMC Psychiatry 21:35. doi: 10.1186/s12888-020-03030-z, PMID: 33435926 PMC7805238

[ref64] Word Health Organization. Optimizing brain health across the life course. (2022) Geneva: WHO position paper. Available at: https://creativecommons.org/licenses/by-nc-sa/3.0/igo/

[ref65] World Health Organization. Global action plan on the public health response to dementia 2017-2025. (2017) Available at: https://iris.who.int/handle/10665/259615

[ref66] Xie M. Pallegar P. N. Parusel S. Nguyen A. T. Wu L. J. (2023). Regulation of cortical hyperexcitability in amyotrophic lateral sclerosis: Focusing on glial mechanisms. Mol. Neurodegener. 18:75. doi: 10.1186/s13024-023-00665-w 37858176 PMC10585818

[ref67] Xu X. Zhao H. Song Y. Cai H. Zhao W. Tang J.. (2024). Molecular mechanisms underlying the neural correlates of working memory. BMC Biol. 22:238. doi: 10.1186/s12915-024-02039-0, PMID: 39428484 PMC11492763

[ref68] Yoshimura N. Tsuda H. Aquino D. Takagi A. Ogata Y. Koike Y.. (2020). Age-related decline of sensorimotor integration influences resting-state functional brain connectivity. Brain Sci. 10, 1–14. doi: 10.3390/brainsci10120966, PMID: 33321926 PMC7764051

[ref69] Zadey S. Buss S. S. McDonald K. Press D. Z. Pascual-Leone A. Fried P. J. (2021). Higher motor cortical excitability linked to greater cognitive dysfunction in Alzheimer’s disease: results from two independent cohorts. Neurobiol. Aging 108, 24–33. doi: 10.1016/j.neurobiolaging.2021.06.007, PMID: 34479168 PMC8616846

[ref70] Zhang N. K. Zhang S. K. Zhang L. I. Tao H. W. Zhang G. W. (2023). Sensory processing deficits and related cortical pathological changes in Alzheimer’s disease. Front. Aging Neurosci. 15:1213379. doi: 10.3389/fnagi.2023.1213379 37649717 PMC10464619

